# ﻿Additions to the *Entoloma* (Agaricales, Entolomataceae) from China: Description of five species with one new to science

**DOI:** 10.3897/mycokeys.123.162577

**Published:** 2025-10-10

**Authors:** Xu Liu, Qiang Chen, Quanju Xiang, Yunfu Gu, Xixi Han, Rui Xing, Ruilin Zhao, Maoqiang He

**Affiliations:** 1 College of Resource Sciences and Technology, Sichuan Agricultural University, Chengdu 611130, China; 2 State Key Laboratory of Mycology, Institute of Microbiology, Chinese Academy of Sciences, Beijing 100101, China; 3 Center of Excellence in Fungal Research, Mae Fah Luang University, Chiang Rai 57100, Thailand; 4 School of Science, Mae Fah Luang University, Chiang Rai 57100, Thailand; 5 Northwest Institute of Plateau Biology,Chinese Academy of Sciences, Xining,Qinghai, China; 6 Qinghai Provincial Key Laboratory of Crop Molecular Breeding, Xining, Qinghai, China; 7 College of Life Sciences, University of Chinese Academy of Sciences, Huairou District, Beijing, 100408, China

**Keywords:** Entolomataceae, new taxa, phylogeny, taxonomy

## Abstract

*Entoloma* is one of the top three genera in species diversity of Agaricomycotina. In this study, five species from Qilian Mountain in China were documented. One new species *E. griseopileum* belonging to subg. Leptonia was introduced based on morphological and phylogenetic analyses. This new species is characterized by its gray basidiomes with fibrillose pileus and wood-decaying lifestyle. Four known species are *E. glaucobasis*, *E. incanum*, and *E. verae* in subg. Cyanula, and *E. fuligineocinereum* in subg. Nolanea. All five species are documented with descriptions, photo plates, and molecular data including ITS, LSU, and mtSSU sequences.

## ﻿Introduction

*Entoloma* (Fr.) P. Kumm., typified by *E. sinuatum* (Bull.) P. Kumm., is one of the top three genera in species diversity of Agaricomycotina. It is estimated that there are more than 1800 species of *Entoloma* worldwide (He et al. 2019a). The general morphological characteristics of *Entoloma*are:basidiomes agaricoid, secotioid or gasteroid; agaricoid habit variable as tricholomatoid, mycenoid, collybioid, clitocyboid, omphalioid or pleurotoid; pink spore print or pinkish brown, lamellae almost free, adnexed to adnate, adnate-emarginate or adnate-decurrent, spores angular in all views which is entirely covered with facets delineated by ridges; clamp-connections often absent; cheilocystidia rare; pileipellis variable (cutis, trichoderm, hymeniderm) with diverse pigmentation(Noordeloos 1981; Largent 1986). Most species of this genus are saprotrophic, but there are also some mycorrhizal or weakly parasitic taxa. For example, members of sections *Entoloma* and *Rhodopolia* are ectomycorrhizal, and Rosaceae-associated members of section Nolanidea are considered weakly parasitic (Agerer and Waller 1993; Tedersoo et al. 2010).

A robust molecular-based taxonomic system for *Entoloma* is not established yet. Traditional taxonomic studies divided *Entoloma* into 13 subgenera based on morphological characteristics, such as basidiome forms, pileipellis, basidiospores, cystidia, and clamp connections (Noordeloos and Gates 2012a; Kaygusuz et al. 2024). Another opinion treated these subgenera as genera (Aime et al. 2010, Karstedt et al. 2020; Niveiro et al. 2021). *Entoloma* constitutes the dominant species diversity within Entolomataceae. The first comprehensive molecular phylogenetic study of Entolomataceae indicated there are two main clades of *Entoloma*, basal and crown clades. The basal clade is equal to Prunuloides clade and crown clade is composed of Rhodopoloid clade, *Inocephalus*-*Cyanula* clade, and *Nolanea*-*Claudopus*. Subgenus Leptonia is found to be polyphyletic and distributed in two different subclades in the crown clade (Co-David et al. 2009).

In the past decades, most taxonomic studies of *Entoloma* are focused on American and European areas while species diversity of *Entoloma* in China is relatively poorly known. In recent years, species diversity of *Entoloma* in China has been explored.There are many new species of *Entoloma* introduced from China based on morphological and molecular data (He et al. 2012, 2013, 2015, 2019b; Chen et al. 2024, 2025). The Qilian Mountains, located between Qinghai and Gansu provinces in northwest China, is a critical biodiversity corridor connecting northern and southern China. During our macrofungi diversity surveys in Qilian Mountains, several notable samples of *Entoloma* were collected. In this study, based on morphological and multigene phylogenetic analyses, we identified and documented five Entoloma species including one new to science in subgenus Leptonia.

## ﻿Materials and methods

### ﻿Morphological examination

Specimens were collected during the rainy season (July to August). Photographs were taken immediately in the field. Basidiomes were wrapped in aluminium foil or put in plastic boxes separately. Macro morphological characteristics and habit information were recorded when specimens were fresh. Every specimen was dried in an electrical food drier at 40 °C, then kept in a plastic ziplock bag and deposited in Herbarium MycologicumAcademiaeSinicae (HMAS). Anatomical and cytological characteristics were observed using dry specimens under the light microscope (SOPTOP EX33). Observed characteristics included basidiospores, basidia, cystidia and pileipellis. At least 20 measurements were made for each characteristic. Measurements were analyzed and recorded as X = the mean of length by width ± SD, Q = the quotient of basidiospore length to width, and Q_m_ = the mean of Q values ± SD. All the protocols of morphological study followed Largent’s methodology (Largent 1986).

### ﻿DNA extraction and PCR

Genomic DNA of each specimen was extracted by abroad-spectrum plant Rapid Genomic DNA Kitfollowed the manufacturer’s protocol. Three genes were applied in the phylogenetic analyses viz. internal transcribed spacer (ITS), large ribosomal subunit (LSU), and mitochondrial ribosomal small subunit (mtSSU). The primers for each gene are ITS4/ITS5, LR5/LROR, and NS1/NS4, respectively. The PCR programs are followed describe in He et al. (2017). The PCR products were sequenced in Sangon Biotech (Shanghai) Co., Ltd. Both directions were sequenced to ensure accuracy of each gene.

### ﻿Phylogenetic analyses

**Figure 1.**
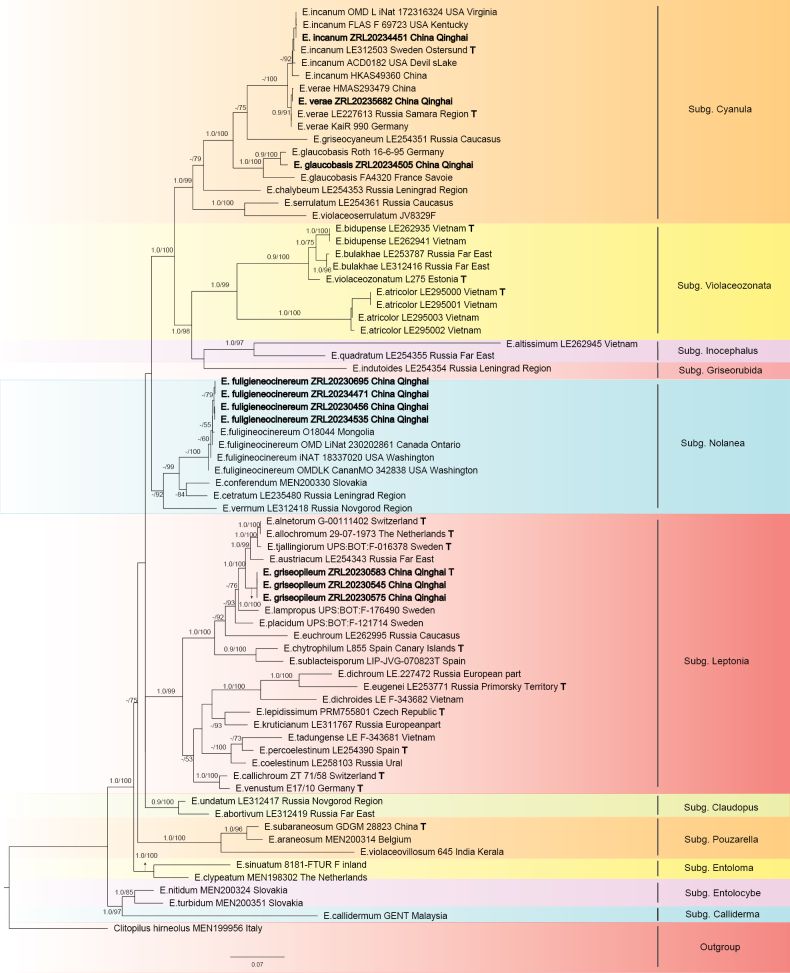
Maximum likelihood tree of *Entoloma* based on ITS, LSU, and mtSSU genes, rooted with *Clitopilus hirneolus*. Bayesian posterior probability (PP) values ≥ 90% or Bootstrap support (BS) values ≥ 50% are indicated at the internodes (PP/BS). New samples are in bold.The type strains cited indicated by a bold “T”.

Information of reference and new generated sequences were listed in Suppl. material 1. New generated sequences were checked in Geneious Prime v.2025.0.3. Alignments were made using Muscle for each gene separately (Edgar 2004), then adjusted by hand to remove the ambiguous region. The dataset was made for genus *Entoloma*. The *Entoloma*dataset includes 72ITS, 53LSU, and 43mtSSU. Maximum likelihood (ML) analysis and bootstrap values calculation were performed in raxmlGUI 1.5b1 with GTRGAMMA model with 1000 replicates (Silvestro and Michalak 2012). ModelFinder v2.2.0 (Kalyaanamoorthy et al. 2017) was used to select the best-fit partition model (Edge-linked) using AIC criterion. In the *Entoloma* dataset, Best-fit model according to AIC are GTR+F+I+G4 for ITS, GTR+F+I+G4 for LSU, and GTR+F+I+G4 for mtSSU. Bayesian Inference (BI) analysis was performed by MrBayes 3.1.2. Ten million generations were run for six Markov chains and sampled every 1000^th^ generation. Burn-ins was determined in Tracer v1.6 with effective sample sizes (ESS) higher than 200 (http://tree.bio.ed.ac.uk/software/tracer). Remaining trees were used to calculate Bayesian posterior probabilities (PP). Maximum likelihood (ML) analysis and bootstrap values calculation were performed in raxmlGUI 1.5b1 with GTRGAMMA model with 1000 replicates (Silvestro and Michalak 2012). Phylogenetic trees were presented in Fig. 1.

## ﻿Phylogenetic results

The ML tree of *Entoloma* is presented in Fig. 1. There are 75 samples involved representing 48 species of *Entoloma* and one outgroup species *Clitopilus hirneolus*. These 48 species represent twelve subgenera of *Entoloma*. Samples collected from Qilian Mountains distributed in three subgenera, viz. subg. Cyanula, subg. Nolanea, subg. Leptonia. In the *Cyanula* clade, three samples from Qilian Mountains clustered in *E. incanum*, *E. verae*,and *E. glaucobasis* clades with full bootstrap values. Four samples clustered in *E. fuligineocinereum* clade in subg. Nolanea with full bootstrap and PP values. Three samples clustered in *Entoloma* and sister to the *E. tallingiorum* with the statistical values PP/BS = 1.0/99.Three Qinghai samples clustered together and sister to the clade composed by *E. austriacum*, *E. alnetorum*,and *E. tjallingiorum* with the statistical values PP/BS = 1.0/100.

## ﻿Taxonomy

### ﻿ Entoloma fuligineocinereum

Taxon classificationFungiAgaricalesEntolomataceae

Mešić & Tkalčec, Phytotaxa 289(3): 297 (2016)

FF3425BA-8FA7-57D6-A4B4-9AC406A4C2E1

Fig. 2

#### Basionym.

*Nolanea latifolia* Kauffman, Pap. Mich. Acad. Sci. 5: 140, 1926 [1925].

#### Description.

Pileus is 23.0–32.2 mm in diameter, convex or broadly convex, occasionally umbonate on the disc, margin entire, occasionally straight; dry orhygrophanous, smooth, background gray or dark brown. *Lamellae* up to 3 mm broad, adnate or slightly sinuate, light brown, brown, getting dark near the pileus edge, eroded,veined. Stipe 72.4–80.2 × 3.2–3.8 mm, concolor with the pileus, paler toward base, cylindrical,equal, surface dry, smooth, silky, occasionally with the white fibrils or punctate near the pileus.

Basidiospores 8.6–11.6 × 8.1–9.8 μm [x̄ =9.6 ± 0.7 × 8.7 ± 0.4 μm, Q = 1.0–1.2, Q_m_ = 1.1 ± 0.1, n = 20], subglobose-angular, asymmetrical, with 5–7 side. Basidia 40.7–47.9 × 11.2–13.2 μm, 4-spored. Cheilocystidia absent. Pileipellis a cutis with a transition to a plagiotrichoderm and a trichoderm of cylindrical to slightly inflated hyphae14.2–28.9 μm wide, with lightmembranous brown pigment. Clamp connections absent. Lamellae edge is fertile.

**Figure 2.**
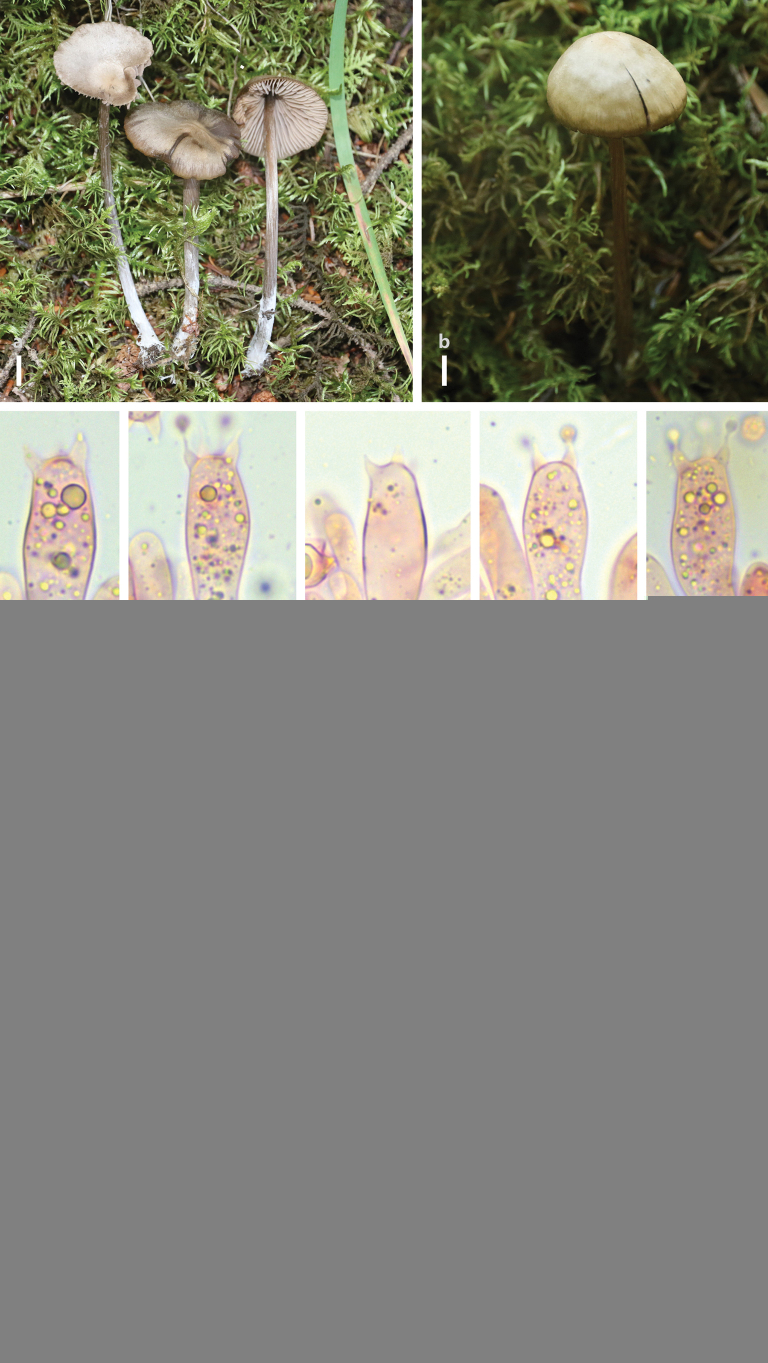
*Entoloma fuligineocinereum*. a, b. Basidiomes in the field; c–g. Basidia; **h, i.** Basidiospores; **j.** Pileipellis hyphae; k, l. Lamellae edge. Scale bars: 5 mm (a, b); 5 μm (c–g); 10 μm (h–I); 40 μm (j); 5 μm (k, l).

#### Notes.

Our samples clustered with samples from Northern America and Mongolia of *E. fuligineocinereum* in the phylogenetic tree (Fig. 1). Morphologically, the morphological characteristics of our samples differs slightly from the previous descriptions. Basidiomes color of our samples is paler than those described in Largent (1971). In addition, our Chinese samples present slightly larger basidiospores compared with the holotype (7.5–9 × 7–8.5 μm, x̄ = 8.3 × 7.7 μm, Largent 1971).

#### Specimen examined.

China • Qinghai province, Qilian Mountain National Park, Sigou, Donghaikou, 9 July 2023, MQ He, *ZRL20230456* (HMAS300882) • Qinghai Province, Haibei Tibetan Autonomous Prefecture, Qilian County, Binggoulinhai, 15 July 2023, MQ He, *ZRL20230695* (HMAS300880) • Qinghai Province, Tongren City, ZamaoKasuhu, 23 August 2023, MQ He, *ZRL20234471* (HMAS300879) • Qinghai Province, Huangnan Tibetan Autonomous Prefecture, Maixiu Forest Farm, 22 August 2023, MQ He, *ZRL20234535* (HMAS300881).

### ﻿ Entoloma glaucobasis

Taxon classificationFungiAgaricalesEntolomataceae

Huijsman ex Noordel., Persoonia 12(4): 260 (1985)

F9449AB8-3839-5AF0-BEA2-E945440F03F8

Fig. 3

#### Description.

Pileus 29–33.4 mm in diameter, campanulate, occasionally with a broad umbo, disc depressed or deeply indented, margin entire, straight, slightly uplifted; surfacedry, background gray or light brown, covered with brown fibrillose scales in triangular shape, scales denser and darker at the disc. *Lamellae* up to 4 mm broad, adnate to decurrent, subdistant, white, light pink, entire, veined. Stipe 48.1–59.6 × 2.3–2.5 cm, light brown, concolor with the pileus, surface dry, smooth, base white with hypha.

Basidiospores 9.3–12.0 × 6.7–9.2 μm [x̄ =10.7 ± 0.7 × 8.1 ± 0.6 μm, Q = 1.2–1.6, Q_m_ = 1.3 ± 0.1, n = 20], heterodiametric, with 5–7 angles in side-view. Basidia 28.5–38.6 × 9.8–13.6 μm, 4-spored. Cheilocystidia basidia-like, 13.2–19.8 × 3.7–7.4 μm.Pileipellis a cutis with a transition to a plagiotrichoderm and a trichoderm of cylindrical to slightly inflated hyphae 8.2–17.9 μm wide, hyaline, membranous light brown. Clamp connections absent. Lamellae edge is heterogeneous.

**Figure 3.**
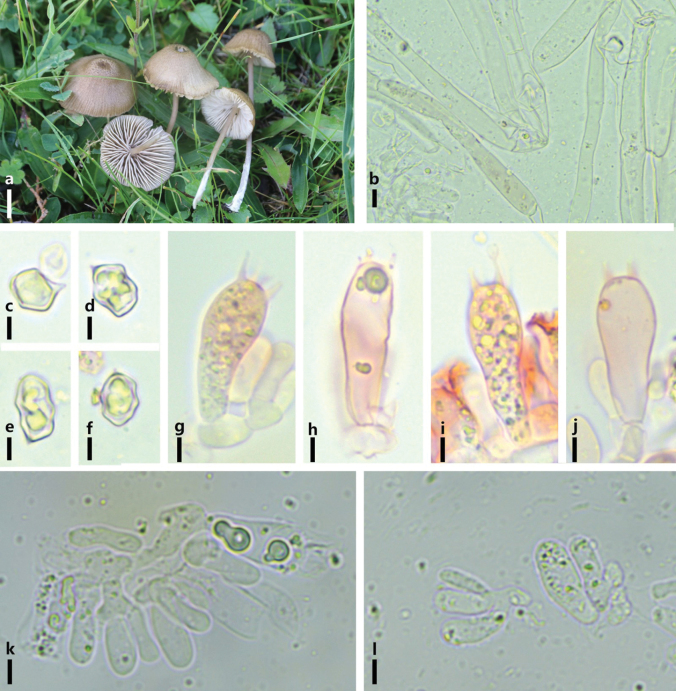
*Entoloma glaucobasis*. a. Basidiomes in the field; b. Pileipellis hyphae; c–f. Basidiospores; g–j. Basidia; k–i. Cheilocystidia. Scale bars: 5 mm (a); 10 μm (b); 5 μm (c–f); 5 μm (g–j); 5 μm (k, l).

#### Notes.

A sample from Qilian Mountain clustered two European samples of *E. glaucobasis*in the phylogenetic tree (Fig. 1). This species is originally described from Germany. Our sample generally coincides with the original description of *E. glaucobasis*. Difference is that the cheilocystidia is basidia-like in our sample while is clavate or vesicular according to the original description (Noordeloos 1985).

#### Specimen examined.

China • Qinghai Province, Huangnan Tibetan Autonomous Prefecture, Maixiu Forest Farm, 22 August 2023, MQ He, *ZRL20234505* (HMAS300878).

### ﻿ Entoloma griseopileum

Taxon classificationFungiAgaricalesEntolomataceae

M.Q. He & X. Liu sp. nov.

6F3D9D09-B798-5DCF-B01B-B4B8E632C88A

Fungal Names: FN 572836

Fig. 4

#### Etymology.

the epithet “*griseo*” means gray, the name refers to the grayish pileus.

#### Holotype.

China • Qinghai Province Mutual Aid County, Beishan National Forest Park,Langshidang, 10 July 2023, MQ He,*ZRL20230583* (HMAS300884).

#### Diagnosis.

This species is characterized by its gray basidiomes and fibrillose pileus.

#### Original description.

Pileus is 26–32 mm in diameter, broadly convex, umbonate on the disc, margin slightly decurved when young, getting straight or slightly uplifted when mature; surface dry, fibrillose, background gray, fibrils light brown, denser and darker on the disc. *Lamellae* up to 3 mm broad, adnate or slightly sinuate, eroded, white, slightly pink, getting brown or dark brown near the pileus edge. Stipe 33.53–42.20 × 3.2–3.5 mm, concolor with the pileus, darker toward base, cylindrical,equal,surface dry, smooth, silky, occasionally with the same fibrils as on pileus.

**Figure 4.**
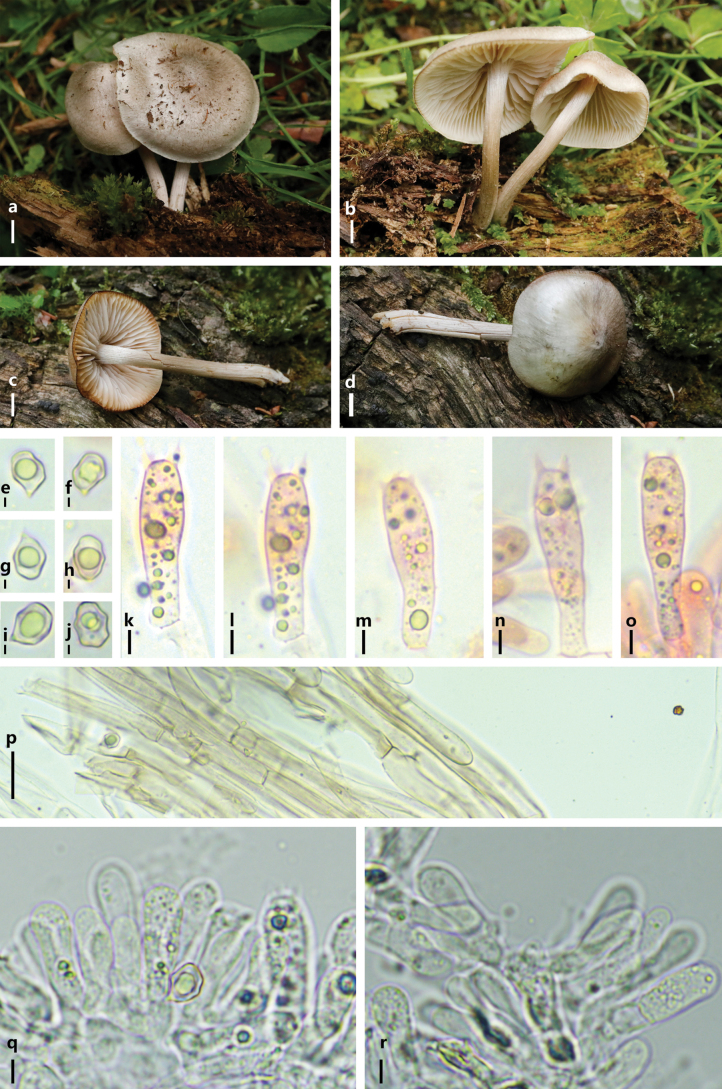
*Entoloma griseopileum*. a–d. Basidiomes in the field; e–j. Basidiospores; k–o. Basidia; p. Pileipellis hyphae; q–r. Lamellae edge. Scale bars: 5 mm (a–d); 2 μm (e–j); 5 μm (k–o); 10 μm (p); 5 μm (q–r).

Basidiospores 6.8–10.1 × 5.9–7.7 μm [x̄ =8.9 ± 0.7 × 6.7 ± 0.4 μm, Q = 1.0–1.6, Q_m_ = 1.3 ± 0.1, n = 20], heterodiametric, with 5–7 angles in side-view. Basidia 31.8–40.6 × 9.3–13.8 μm, 4-spored. Cheilocystidia absent. Pileipellis a cutis with a transition to a plagiotrichoderm and a trichoderm of cylindrical to slightly inflated hyphae 4.6–11.1 μm wide, with membranous brown pigment. Clamp connections absent. Lamellae edge is fertile.

#### Habitat.

Wood-inhabiting.

#### Notes.

Morphologically, *E. griseopileum* resembles *E. bisporigerum* in the field which also can be found in northern China (Li et al. 2015). Both species have grayish small basidiomes (pileus smaller than 4cm in diameter). But under microscope, *E. bisporigerum* can be distinguished from *E. griseopileum* by its larger basidiospores (11.5–13.5 × 7.5–9 μm). Furthermore, *E. bisporigerum* grows on soil, while *E. griseopileum* grows on wood.

#### Other specimens examined.

China • Qinghai Province, Beishan National Forest Park, Langshidang, 10 July 2023, MQ He, *ZRL20230545* (HMAS300885), *ZRL20230575* (HMAS300883).

### ﻿ Entoloma incanum

Taxon classificationFungiAgaricalesEntolomataceae

(Fr.) Hesler,Beih. Nova Hedwigia 23: 147 (1967)

102F6687-305C-5753-835F-DD1938894CA0

Fig. 5

#### Description.

Pileus is 22.9–26.0 mm in diameter, broadly convex, disc depressed to deeply indented, margin entire;surfacesmooth or slightly with white fibrils, brown, yellowish brown, darker at the disc, getting paler or yellower towards the edge. *Lamellae* up to 3 mm broad, adnate to decurrent, subdistant, white, light yellow,entire,veined. Stipe 53.6 × 6.6 mm, yellow, or light greenish yellow, hollow,compressed, surface dry, smooth, base white with hypha.

Basidiospores 9.8–13.6 × 6.3–8.4 μm [x̄ =11.6 ± 1.1 × 7.4 ± 0.6 μm, Q = 1.3–2.0, Q_m_ = 1.6 ± 0.2, n = 20], heterodiametric, with 6–8 angles in side-view. Basidia 40.3–60.8 × 10.2–15.9 μm, 4-spored. Cheilocystidia absent. Pileipellis a cutis with a transition to a plagiotrichoderm and a trichoderm of cylindrical to slightly inflated hyphae 6.0–15.5 μm wide, with membranous light brown pigment. Clamp connections absent. Lamellae edge is fertile.

**Figure 5.**
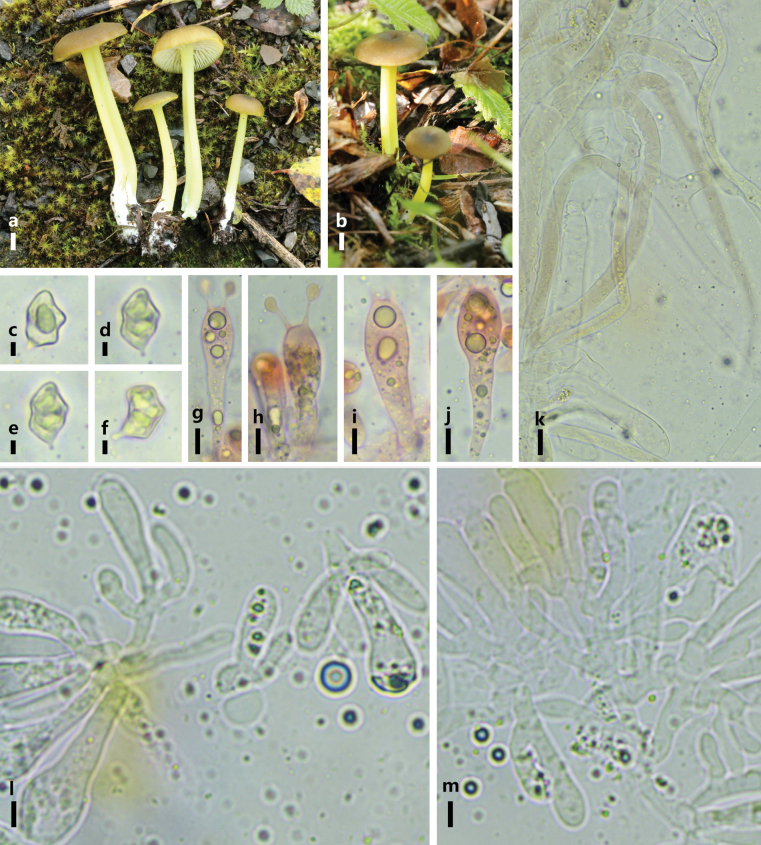
*Entoloma incanum*. a, b. Basidiomes in the field; c–f. Basidiospores; g–j. Basidia; k. Pileipellis hyphae; i–m. Lamellae edge. Scale bars: 5 mm (a, b); 2 μm (c–f); 10 μm (g–j); 20 μm (k); 2 μm (l, m).

#### Notes.

Our Chinese samples clustered with northern America samples of *E. incanum* in the phylogenetic tree with full statistical values (Fig. 1). *Entoloma incanum* is a remarkable species in the field due to its greenish-brown or yellowish-brown basidiomes with a bright stipe in green or yellow. Our samples present a relatively browner pileus than American samples (Kuo 2013).

#### Specimen examined.

China • Qinghai Province, Tongren City, Zhamao Village, 23 August 2023, MQ He, *ZRL20234451* (HMAS300876).

### ﻿ Entoloma verae

Taxon classificationFungiAgaricalesEntolomataceae

O.V. Morozova, Noordel., Reschke, F. Salzmann & Dima, Persoonia 47: 307 (2021)

FBD55E7A-5B19-53B1-A1F8-00542E55FD20

Fig. 6

#### Description.

Pileus 21.4 mm in diameter, plane, disc deeply indented, margin entire, surfacedry, with distinct radial dark brown fibrils on pale background,sometimes cracked when dry or old, pileus edge dark brown. *Lamellae* up to 4 mm broad, adnate to decurrent, subdistant, yellowish white and light pink,entire,veined. Stipe 36.4 × 2.2 cm, green, yellowish green,equal, surface dry, with yellow tiny fibrils, base white with hypha, staining bright greenish blue when bruised.

**Figure 6.**
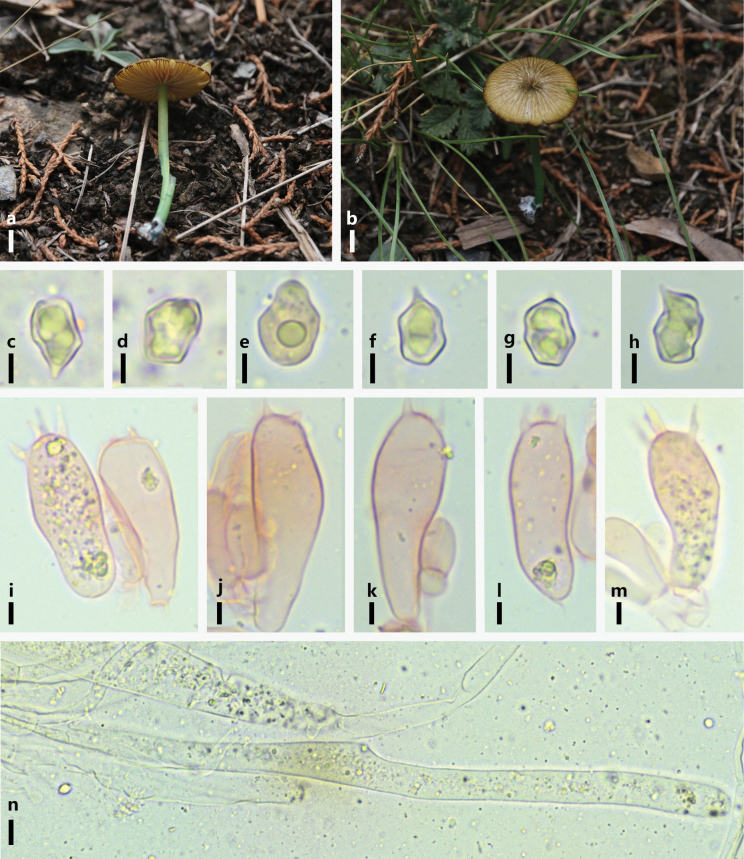
*Entoloma verae*. a, b. Basidiomes in the field; c–h. Basidiospores; i–m. Basidia; n. Pileipellis hyphae. Scale bars: 5 mm (a, b); 5 μm (c–h); 5 μm (i–m); 10 μm (n).

Basidiospores 8.9–12.5 × 5.8–8.0 μm [x̄ = 10.9 ± 0.9 × 7.2 ± 0.5 μm, Q = 1.2–1.8, Q_m_ = 1.5 ± 0.1, n = 20], heterodiametric, with 6–7 angles in side-view. Basidia 31.6–43.4 × 12.4–17.4 μm, 4-spored. Cheilocystidia not observed. Pileipellis a cutis with a cylindrical to slightly inflated hyphae 4.5–14.5 μm wide, hyaline. Membranous pigment. Clamp connections absent.

#### Notes.

Our sample clustered with European samples including the type of *E. verae* in the phylogenetic tree with full statistical values. Morphological characteristics of our sample generally coincides with the original description. Small differences are pileus color and basidiospore size. The pileus of our sample is darker and the basidiospore is smaller compared to the original description (Crous et al. 2021).

#### Specimen examined.

China • Qinghai province, Haidong City, Tu Autonomous County of Huzhu, Ganchan Temple, 26 August 2023, MQ He, *ZRL20235682* (HMAS300877).

## ﻿Conclusions

In this study, five *Entoloma* species were documented from China. These include one new species, *E. griseopileum*in subg. Leptonia. The new species is characterized by its gray basidiomes, fibrillose pileus, and wood-decaying habit. The other four known species are *E. glaucobasis*, *E. incanum*, and *E. verae* in subg. Cyanula, and *E. fuligineocinereum* in subg. Nolanea. All five species are found in the Qilian Mountains. The macrofungal diversity of this region has been explored in recent years, and many new species, for example, from *Agaricus*, *Cystoderma*, and *Lyophyllum*, have been found and documented (Zhang et al. 2017; Li et al. 2021; Wei et al. 2023). We assumed that there would be continuous descriptions of species from this region as it is part of the Qinghai-Xizang Plateau and is a critical biodiversity corridor connecting northern and southern China.

## Supplementary Material

XML Treatment for Entoloma fuligineocinereum

XML Treatment for Entoloma glaucobasis

XML Treatment for Entoloma griseopileum

XML Treatment for Entoloma incanum

XML Treatment for Entoloma verae

## ﻿Additional information

### Use of AI

No use of AI was reported.

### Author ORCIDs

Ruilin Zhao https://orcid.org/0000-0001-8129-9339

Maoqiang He https://orcid.org/0000-0002-9300-7484

### Data availability

All of the data that support the findings of this study are available in the main text or Supplementary Information.

## Supplementary materials

Supplementary material 1 Sequeneces information used in the phylogenetic analysis Data type xlsx https://binary.pensoft.net/file/1435472This dataset is made available under the Open Database License (http://opendatacommons.org/licenses/odbl/1.0/). The Open Database License (ODbL) is a license agreement intended to allow users to freely share, modify, and use this Dataset while maintaining this same freedom for others, provided that the original source and author(s) are credited.Xu Liu, Qiang Chen, Quanju Xiang, Yunfu Gu, Xixi Han, Rui Xing, Ruilin Zhao, Maoqiang He
